# A panel of miRNAs as prognostic markers for African-American patients with triple negative breast cancer

**DOI:** 10.1186/s12885-021-08573-2

**Published:** 2021-07-27

**Authors:** Safaa Turkistani, Bruna M. Sugita, Paolo Fadda, Rafael Marchi, Ali Afsari, Tammey Naab, Victor Apprey, Robert L. Copeland, Michael C. Campbell, Luciane R. Cavalli, Yasmine Kanaan

**Affiliations:** 1 Department of Microbiology, Howard University Cancer Center, Howard University, Washington DC, USA; 2 Research Institute Pelé Pequeno Príncipe, Faculdades Pequeno Príncipe, Curitiba, PR Brazil; 3 Genomics Shared Resource, Comprehensive Cancer Center, The Ohio State University, Columbus, OH USA; 4 Department of Pathology, Howard University Hospital, Washington DC, USA; 5 Department of Community and Family Medicine, Howard University, Washington DC, USA; 6 Department of Pharmacology, College of Medicine and Cancer Center, Howard University, Washington DC, USA; 7 Department of Biology, Howard University, Washington DC, USA; 8 Department of Oncology, Lombardi Comprehensive Cancer Center, Georgetown University, Washington DC, USA

**Keywords:** microRNA, Triple-negative breast cancer, African-American, Prognosis, Tumor size, Lymph node

## Abstract

**Background:**

To investigate the global expression profile of miRNAs, their impact on cellular signaling pathways, and their association with poor prognostic parameters in African-American (AA) patients with triple negative breast cancer (TNBC).

**Methods:**

Twenty-five samples of AA TNBC patients were profiled for global miRNA expression and stratified considering three clinical-pathological parameters: tumor size, lymph node (LN), and recurrence (REC) status. Differential miRNA expression analysis was performed for each parameter, and their discriminatory power was determined by Receiver Operating Characteristic (ROC) curve analysis. KMplotter was assessed to determine the association of the miRNAs with survival, and functional enrichment analysis to determine the main affected pathways and miRNA/mRNA target interactions.

**Results:**

A panel of eight, 23 and 27 miRNAs were associated with tumor size, LN, and REC status, respectively. Combined ROC analysis of two (miR-2117, and miR-378c), seven (let-7f-5p, miR-1255b-5p, miR-1268b, miR-200c-3p, miR-520d, miR-527, and miR-518a-5p), and three (miR-1200, miR-1249-3p, and miR-1271-3p) miRNAs showed a robust discriminatory power based on tumor size (AUC = 0.917), LN (AUC = 0.945) and REC (AUC = 0.981) status, respectively. Enrichment pathway analysis revealed their involvement in proteoglycans and glycan and cancer-associated pathways. Eight miRNAs with deregulated expressions in patients with large tumor size, positive LN metastasis, and recurrence were significantly associated with lower survival rates. Finally, the construction of miRNA/mRNA networks based in experimentally validated mRNA targets, revealed nodes of critical cancer genes, such as *AKT1*, *BCL2*, *CDKN1A*, *EZR* and *PTEN*.

**Conclusions:**

Altogether, our data indicate that miRNA deregulated expression is a relevant biological factor that can be associated with the poor prognosis in TNBC of AA patients, by conferring to their TNBC cells aggressive phenotypes that are reflected in the clinical characteristics evaluated in this study.

**Supplementary Information:**

The online version contains supplementary material available at 10.1186/s12885-021-08573-2.

## Background

Breast cancer is one of the most frequent types of female cancer worldwide and one of the main causes of death in women [1]. Several new target therapies are currently in development with significant potential to reduce mortality in patients with varied breast cancer subtypes by significantly increasing their overall survival [2]. However, for triple negative breast cancer (TNBC), there are still few approved targeted therapies. Inhibitors of the programmed death-ligand 1 (PDL1) have been recently approved for the treatment of unresectable locally advanced or metastatic TNBC patients. This targeted therapy is, however, only beneficial for patients with PDL1 positive tumor expression [3, 4].

TNBC is considered one of the most aggressive subtypes of breast cancer, with high rates of progression and poor prognosis [5]. Comprehensive molecular-based studies have been extensively performed to identify new biomarkers that can be used for diagnosis, prognosis, and more efficient treatment regimens for this tumor subtype. One class of biomarkers that has emerged with promising therapeutic potential is the non-coding microRNAs (miRNAs) [6, 7]. With the technological advances in nanoparticle delivery systems, this class of biomarkers has been shown to be effective in inhibiting tumor progression and metastasis in several tumor models, including TNBC [8–10].

MiRNAs are conserved endogenous small RNA molecules that can regulate a range of developmental and physiological processes in the cells, in a tissue-specific manner [11]. MiRNAs expression alterations are associated with the development of pathological processes and chronic diseases [12]. In cancer, miRNAs play a critical role in tumor initiation and progression, through their regulatory role in the expression of gene targets involved in multiple signaling pathways, such as cell proliferation, differentiation, and cell death [13–17]. In TNBC, many miRNAs with deregulated expression have been identified and associated with aggressive cancer phenotypes, including larger tumor sizes, early tumor recurrence, lymph node, and metastatic invasion, and lower survival rates [18].

Several studies have shown that miRNAs present variable expression patterns according to race and/or ethnic groups [19–23]. However, few of them were conducted in patients’ tumor samples. We have previously shown in genomically characterized African-American (AA) and Latina patients with TNBC, differential patterns of tumor miRNA expression when compared to TNBC patients of European descent [24, 25]. These profiles were shown to be associated with distinct signaling pathways, commonly linked to poor prognosis and shorter survival. Although there are a limited number of studies evaluating the somatic miRNA expression in tumors of genomically ancestral characterized populations, this data supports the relevance of miRNAs as biological contributors to cancer disparities.

In this study, the main objective was to characterize the global patterns of miRNA expression, and their impact on cellular signaling pathways in the TNBC tissue specimens of AA patients. The miRNA patterns were also evaluated according to three selected clinical-pathological parameters: tumor size, lymph node (LN), and recurrence (REC) status.

## Material and methods

### Samples collection, clinical and demographic information

Formalin-fixed paraffin-embedded tissue blocks (FFPE) of 25 TNBC patients were collected from the surgical pathology archives of Howard University Hospital, Washington DC. Clinical and histopathological data were obtained for the patients in a codified manner according to the approved institutional IRB protocols and included age at diagnosis, histologic type, tumor size, Nottingham Histologic Score and Grade [26], lymph node (LN), recurrence (REC), and distant metastasis status. This study was approved by the Office of Regulatory Research Compliance – Institutional Review Board of Howard University (IRB-16-MED-39).

Clinical and histopathological data of the variables above were retrieved for all the cases analyzed. Most of the patients (81.8%) were diagnosed with infiltrating ductal carcinoma, with a mean age at diagnosis of 55.27 ± 9.96 years old (range 32 to 78 years old). All tumors, but one (grade 2), were classified as histological high grade (grade 3). Wide variability in tumor size was observed, with tumors presenting their largest dimension between 1.1 and 22 cm in size (average 5.86 ± 5.17 cm). Ten patients (62.5%) presented tumors smaller than 5 cm, while six patients (37.5%) presented tumors larger than 5 cm. LN involvement (LN+) was present in ten patients (47.6%) affecting one or more LNs while eleven patients (53.4%) were LN negative (LN-). Sixty-eight percent of the patients became disease-free after treatment and had no recurrence, while 32% relapsed (with local and/or distant metastasis) after the selected treatment regimen or never reached a disease-free condition since diagnosis (Table S1).

The classification of the TNBC phenotype was confirmed by immunohistochemistry analysis using estrogen (ER), progesterone (PR), and human epidermal growth factor receptor 2 (HER2) receptor markers, following current guidelines [27, 28].

The AA race information of the patients was initially obtained from self-reported medical records and later confirmed by genotyping analysis for a subset of the patients. The genotyping was performed using the SNP chip Illumina Infinium QC Array (Illumina Inc., CA), which contains 15,949 markers, including, 3000 ancestral informative markers (AIMs). The genotype calling was performed as we previously described [24, 25], using GenomeStudio Software v. 2011.1. Genotypes from the mitochondrial genome and on sex chromosomes were excluded, as well as genotypes with a call rate < 98%. The remaining autosomal genotypes (8687 in total) were integrated with the variant calls from ≥1900 individuals originating from 21 diverse populations in the 1000 Genomes Project. To explore population structure among individuals, Principal Component Analysis (PCA) was conducted on the genome-wide autosomal loci. First, a genetic relationship matrix between pairs of individuals (GRM files) was generated with the GCTA software [29]. Then, using the GRM files as input, the PCA method implemented in GCTA was applied. In these analyses, the default setting (*n* = 20) was accepted, which outputted the first 20 eigenvectors and all of the eigenvalues. Lastly, the top two Principal Components, PC1, and PC2 were plotted using RStudio [30]. PC1 (accounting for 69.1% of the extracted variation) broadly distinguished individuals of African descent from non-Africans, suggesting some genetic distance between African and non-African populations consistent with previous studies of autosomal loci [31, 32] (Figure S1).

### RNA isolation and global miRNA expression analysis

The FFPE tumor specimens were evaluated by a pathologist for the presence of at least 80% of tumor cells. The selected tumor areas were microdissected from unstained 10 μm FFPE tissue sections and total RNA was isolated using TRIzol (Invitrogen Carlsbad, CA, USA) after deparaffinization with Xylene solution. RNA concentration and quality were tested by measuring 260/280 and 260/230 ratios using Nanodrop 2000 spectrophotometer (Willington, DE, USA).

MiRNA expression analysis was performed using Nanostring nCounter Human v3a miRNA Expression Assay (Seattle, WA, USA) that contains human probes derived from miRBase version 22 (http://www.mirbase.org) targeting 827 human miRNAs, six positive controls, eight negative controls, three ligation positive controls, three ligation negative controls, five internal reference genes (*ACTB, B2M, GAPDH, RPL19,* and *RPL0*), and five spike-in controls (ath-miR-159a, cel-miR-248, cel-miR-254, osa-miR-414, and osa-miR-442). Raw data were pre-processed using Nanostring’s Counter RCC collector and normalized using Nanostring nSolver 4.0 software (Background subtraction: the geometric mean of Negative Controls; Technical normalization: the geometric mean of Positive Controls; Codeset content normalization: all genes geometric mean). Normalized data was Log2 transformed and analyzed using the MultiExperiment Viewer software (MeV 4.9.0), GraphPad Prism 8.3.0, and IBM SPSS Statistics 25.

### Differential miRNA expression analysis among the TNBC clinical groups

The tumor samples profiled for miRNA were stratified in different clinical groups according to three selected clinical-pathological parameters: tumor size (≥5 cm or < 5 cm), LN status [positive (LN+) or negative (LN-) and REC status (positive (REC+) or negative (REC-)]. Differential miRNA expression analysis was performed for each of these parameters, resulting in three distinct miRNA panels (Welch-based *t-*Test, *p* ≤ 0.05). Log2 Fold Change (Log2FC) was calculated for each differentially expressed miRNA. Unsupervised and Supervised Hierarchical Clustering analysis was performed using Pearson’s correlation coefficient and average linkage and visualized as heatmaps with a 3-color scheme blue-black-yellow representing values below the median-median-above median, respectively.

### Receiver operating characteristic (ROC) curve analysis

The discriminatory power of the differentially expressed miRNAs between the different clinical groups was tested by constructing the ROC curve and calculating the Area Under the Curve (AUC). Sensitivity was plotted against specificity (%) for the binary classifiers (≥5 cm vs < 5 cm, LN+ vs LN-, and REC+ vs REC-). An AUC of 1.0 (100%) denotes perfect discrimination by the miRNA, whereas an AUC of 0.5 (50%) denotes a complete lack of discrimination by the miRNA. AUCs and 95% corresponding confidence intervals (CI) were calculated for each miRNA using GraphPad Prism v 8.3.0 and IBM SPSS Statistics 25.

Multiple binary logistic regression analysis was used to determine the effect of the panel of miRNAs in discriminating the samples according to their tumor size, LN, and REC status. The most robust miRNA panel combination was also determined for each clinical parameter studied.

### Kaplan Meier (KM) plot analysis

The miRpower KM Plotter Tool [33] was used to calculate hazard ratios, confidence intervals, and log-rank *P* values for the miRNAs that were shown to present a high discriminatory power in each clinical group evaluated. This analysis was performed in relation to survival in the aggregated breast cancer clinical studies extracted from The Cancer Genome Atlas (TCGA) and Molecular Taxonomy of Breast Cancer International Consortium (METABRIC) databases (extraction based on TNBC cases in general, and TNBC cases with known LN status).

### Functional enrichment pathways analysis

The Diana miRPath v.3.0 [34] was used to identify the top signaling pathways that were affected by the significant differentially expressed miRNAs, which is based on the online tool Kyoto Encyclopedia of Genes and Genomes (KEGG) annotation. For these analyses, micro-T-CDS (v5.0) was used to identify the predicted gene targets regulated by the selected miRNAs, applying the *p*-value of 0.05 and a microT threshold of 0.8.

The miRpathDB v.2.0 [35] was used to determine the potential influence of the selected miRNAs in the Integrated Breast Cancer pathway (WP1984) from Wikipathways [36], considering experimentally validated and predicted target genes. This pathway presents relevant proteins and their interactions in breast cancer and has been widely used in miRNA prediction analyses [37, 38].

### In silico functional analysis

The databases miRTarBase [39] and miRnet [40] were used to determine interactions between the selected miRNAs and target genes validated based on strong (reporter assays, Western Blot, and qPCR) and less strong experimental assays (microarray, NGS, pSilac). MirTargetLink Human online software was used to visualize miRNA-mRNA interaction networks [41].

The STRING v.11 database [42] was used to verify protein-protein interactions (PPI) between the validated target genes of each group, applying the minimum interaction score of 0.9 (highest confidence). Cytoscape v.3.8.0 [43] was used to construct molecular interaction networks of selected miRNAs and target genes.

## Results

### Differential miRNA expression analysis among the TNBC clinical groups

**Fig. 1:**
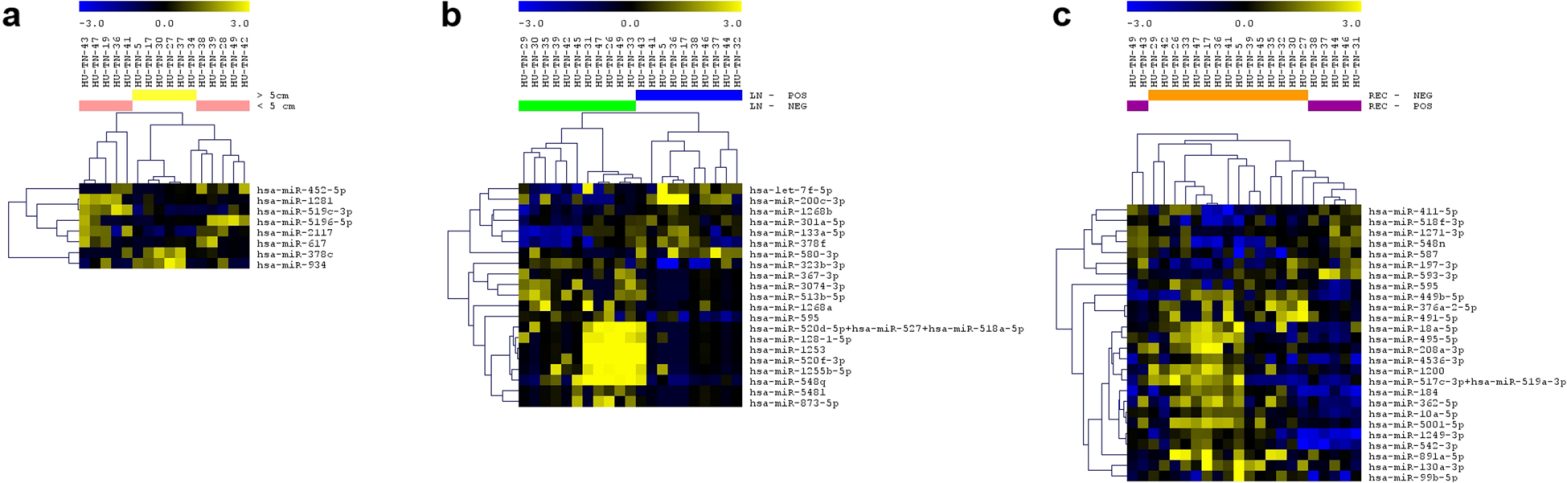
Differentially expressed miRNAs in the analysis of the TNBC cases distributed by tumor size (**a**), LN (**b**) and REC (**c**) status

A total of 25 cases were successfully profiled for the expression of global miRNAs. The analysis of miRNAs expression was performed separately for each of the three selected clinical groups (tumor size, LN, and REC status) (Table 1, Fig. 1). In the analysis of the cases based on tumor size, eight miRNAs were observed differentially expressed between the large (≥5 cm) and small (< 5 cm) tumors, from which two miRNAs were upregulated and six downregulated in the large tumor size cases (*t*-Test *p* < 0.05, Fig. 1a). In the LN group analysis, 23 miRNAs were observed differentially expressed between the LN+ and LN- cases, seven upregulated and 14 downregulated in the LN+ cases (*t*-Test *p* < 0.05, Fig. 1b). Finally, 27 miRNAs were observed differentially expressed between REC+ and REC- cases, seven upregulated and 19 downregulated in the REC+ cases (*t*-Test, p < 0.05, Fig. 1c).

**Table 1:** Log2FC, *p*-value, AUC and corresponding 95%CI for each differentially expressed miRNA observed in the tumor size, LN and REC status groups

**Tumor size (**≥**5 cm/< 5 cm)**	**Log2FC**	***p*-value**	**AUC**	**95% CI**
miR-1281	− 0.87487	0.0479	0.6833	0.4101 to 0.9565
miR-2117	−1.51124	0.0026	0.875	0.6826 to 1.0000
miR-378c	1.24370	0.0457	0.8083	0.5528 to 1.0000
miR-452-5p	−0.81783	0.0329	0.7000	0.4376 to 0.9624
miR-5196-5p	−1.27827	0.0132	0.7500	0.5050 to 0.9950
miR-519c-3p	−1.27906	0.0187	0.8000	0.5804 to 1.0000
miR-617	−0.82711	0.0402	0.7167	0.4536 to 0.9797
miR-934	1.610418	0.0193	0.8333	0.6295 to 1.0000
**LN status**	**Log2FC**	***p*-value**	**AUC**	**95% CI**
let-7f-5p	1.308153	0.0345	0.8091	0.6141 to 1.000
miR-1253	−2.23171	0.0436	0.7909	0.5984 to 0.9834
miR-1255b-5p	−1.43984	0.0298	0.8091	0.6201 to 0.9981
miR-1268a	−0.9362	0.0441	0.7682	0.5632 to 0.9732
miR-1268b	0.7279	0.0084	0.8636	0.6882 to 1.000
miR-128-1-5p	−1.49312	0.0254	0.7818	0.5838 to 0.9799
miR-133a-5p	1.097722	0.0369	0.6727	0.4348 to 0.9106
miR-200c-3p	2.602164	0.0187	0.8182	0.6375 to 0.9988
miR-301a-5p	0.583352	0.0212	0.8091	0.6170 to 1.000
miR-3074-3p	−0.85799	0.0301	0.7273	0.4976 to 0.9570
miR-323b-3p	−1.30263	0.0324	0.7636	0.5366 to 0.9907
miR-367-3p	−0.653	0.0438	0.7545	0.5444 to 0.9647
miR-378f	1.143995	0.0356	0.8000	0.6058 to 0.9942
miR-513b-5p	−1.04378	0.0115	0.7864	0.5790 to 0.9938
miR-520d-5p + miR-527 + miR-518a-5p	−1.53273	0.0161	0.8364	0.6645 to 1.000
miR-520f-3p	−1.7368	0.0355	0.7909	0.5852 to 0.9966
miR-548 l	−0.59929	0.0187	0.7909	0.5936 to 0.9882
miR-548q	−1.98804	0.0105	0.7727	0.5600 to 0.9855
miR-580-3p	1.266989	0.0094	0.8045	0.5962 to 1.000
miR-595	−1.17619	0.0070	0.8182	0.6281 to 1.000
miR-873-5p	−0.86919	0.0434	0.7045	0.4769 to 0.9321
**REC status**	**Log2FC**	***p*-value**	**AUC**	**95% CI**
miR-10a-5p	−0.86382	0.022	0.7905	0.5801 to 1.000
miR-1200	−1.55119	0.0004	0.8571	0.7004 to 1.000
miR-1249-3p	−1.54646	0.0211	0.8286	0.6493 to 1.000
miR-1271-3p	0.962012	0.0108	0.9143	0.7968 to 1.000
miR-130a-3p	−1.45528	0.0144	0.7810	0.5850 to 0.9769
miR-184	−2.01337	0.0005	0.8857	0.7442 to 1.000
miR-18a-5p	−1.2983	0.0226	0.7667	0.5637 to 0.9696
miR-197-3p	1.067639	0.0412	0.7714	0.5689 to 0.9740
miR-208a-3p	−0.94776	0.0372	0.7333	0.5162 to 0.9505
miR-362-5p	−1.31405	0.0486	0.7619	0.5401 to 0.9837
miR-376a-2-5p	−1.07002	0.0055	0.7048	0.4876 to 0.9220
miR-411-5p	1.185286	0.0084	0.7810	0.5876 to 0.9743
miR-449b-5p	−1.76057	0.0003	0.8571	0.6997 to 1.000
miR-4536-3p	−1.25336	0.0292	0.8000	0.6039 to 0.9961
miR-491-5p	−0.97535	0.0373	0.6952	0.4761 to 0.9144
miR-495-5p	−1.18756	0.0291	0.7238	0.5092 to 0.9385
miR-5001-5p	−1.12657	0.0165	0.7905	0.5968 to 0.9841
miR-517c-3p + miR-519a-3p	−1.31722	0.0364	0.6952	0.4759 to 0.9146
miR-518f-3p	1.233145	0.003	0.8286	0.6538 to 1.000
miR-542-3p	−1.47531	0.0253	0.8095	0.6106 to 1.000
miR-548n	1.427446	0.0044	0.8619	0.6799 to 1.000
miR-587	1.032396	0.0112	0.7714	0.5633 to 0.9796
miR-593-3p	1.172516	0.0446	0.8190	0.6257 to 1.000
miR-595	−1.00655	0.0136	0.8381	0.6600 to 1.000
miR-891a-5p	−1.23041	0.0142	0.7619	0.5570 to 0.9668
miR-99b-5p	−1.3409	0.0267	0.8286	0.6574 to 0.9998

**Fig. 2:**
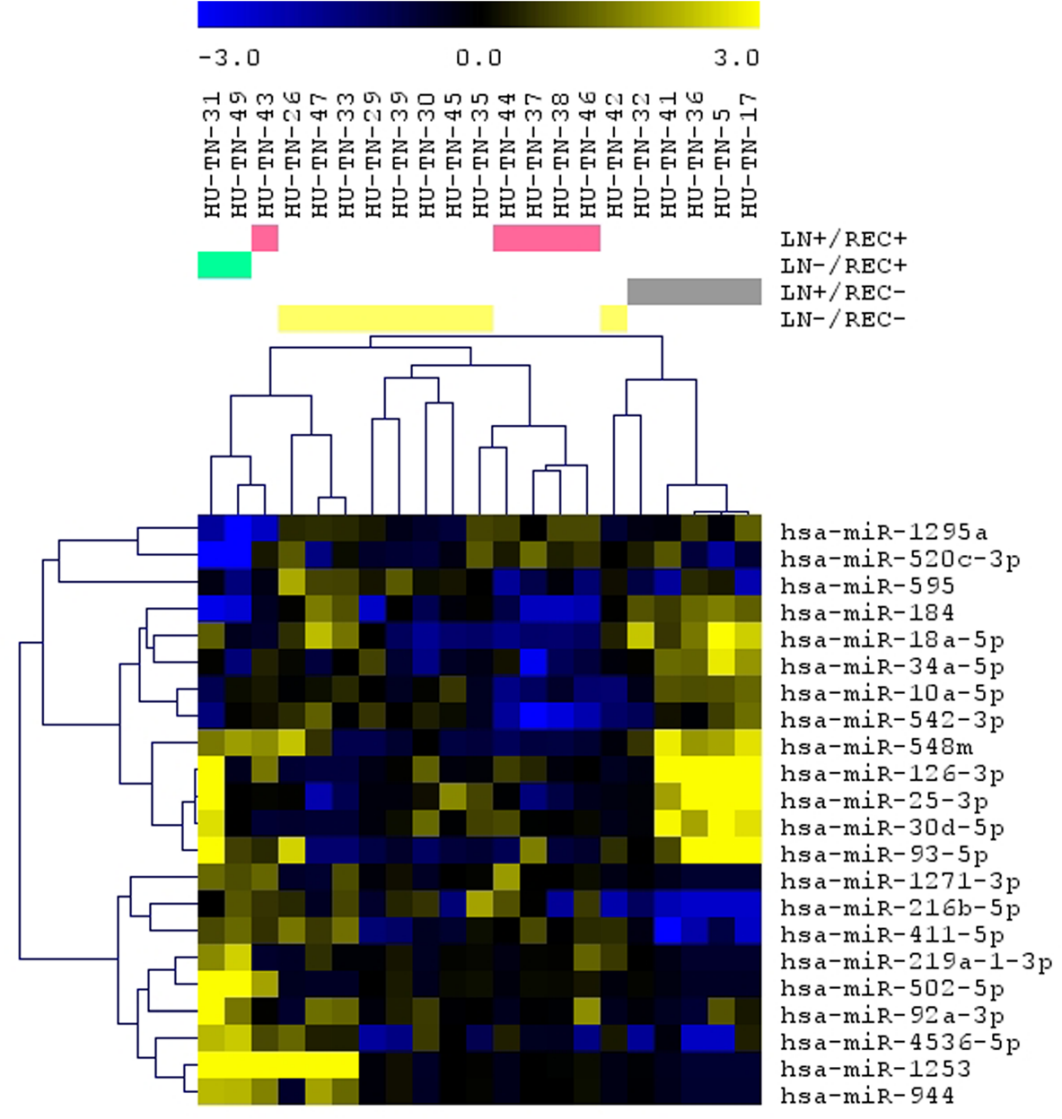
Differentially expressed miRNAs in the analysis of the TNBC cases distributed in the four combined LN and REC status groups

MiRNA differential expression analysis was also performed based on the four combined LN and REC status groups, considering the representative number of patients in each of these sub-groups (especially for the LN+/REC- which represented 23.8% of the patients): LN+/REC+, LN−/REC+, LN+/REC- and LN−/REC- (ANOVA, *p* < 0.01). Twenty-two miRNAs were found differentially expressed among these groups (Fig. 2). The comparison of the differentially expressed miRNAs among the LN, REC and combined LN/REC status groups resulted in eight miRNAs: miR-10a-5p, miR-1271-3p, miR-184, miR-18a-5p, miR-411-5p, and miR-542-3p present in the REC and LN/REC comparisons, and miR-1253 present in LN and LN/REC comparisons, and miR-595 present in all the three comparisons.

### ROC analysis

**Fig. 3:**
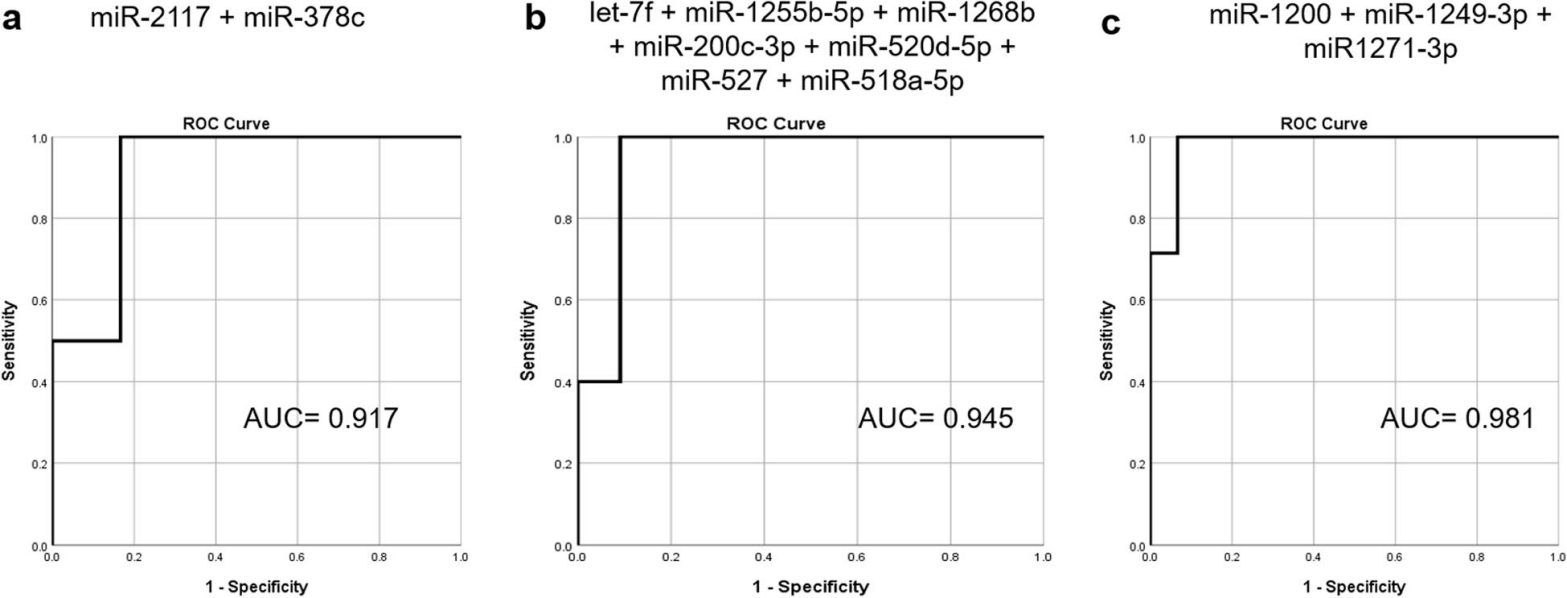
Combined ROC analysis for the most robust discriminatory miRNA panels for tumor size (**a**), LN (**b**) and REC (**c**) status

ROC analysis was performed for each miRNA in each of the clinical groups evaluated. The analysis of the eight miRNAs observed differentially expressed according to tumor size, resulted in AUC values from 0.683 (miR-1281) to 0.875 (miR-2117). Four miRNAs presented AUC higher than 0.800 and were used to determine the most robust miRNA panel for classifying the samples based on tumor size (≥5 cm or < 5 cm). As a result, the combination of miR-2117 and miR-378c showed the highest discriminatory power with an AUC value of 0.917(Fig. 3a).

Eleven out of the 23 differentially expressed miRNAs according to the LN status presented AUC ≥ 0.800, and the combination of let-7f-5p, miR-1255b-5p, miR-1268b, miR-200c-3p, miR-520d-5p + miR-527 + miR-518a-5p expression levels presented the highest discriminatory power (AUC = 0.945; Fig. 3b).

Finally, the ROC analysis of miRNAs differentially expressed according to REC status, showed an AUC ranging from 0.695 (miR-491-5p and miR-517c-3p + miR-519a-3p) to 0.914 (miR-1271-3p). Twelve miRNAs presented AUC values higher than 0.800, and a combined ROC analysis indicates that the most robust miRNA panel consisted of miR-1200, miR-1249-3p and miR-1271-3p (AUC = 0.981; Fig. 3c). The individual AUC values for all the miRNAs differentially expressed per clinical group and their corresponding 95% CI are presented in Table 1.

### Association of the Target miRNA genes with survival using KM plot database

**Fig. 4:**
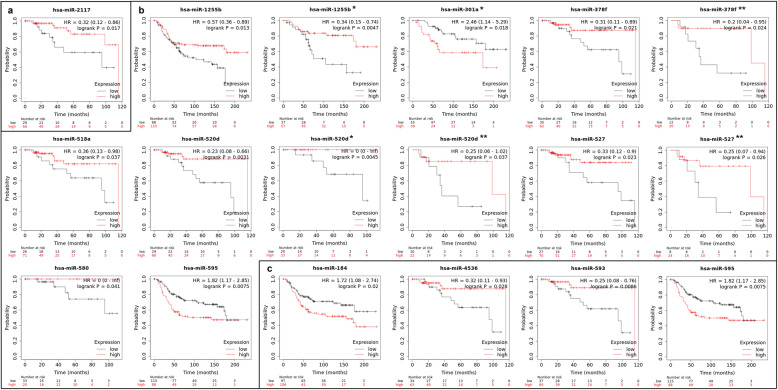
KM Plot graphics of the miRNAs that presented AUC > 0.8 in each clinical group analyzed (**a** tumor size, **b** LN, and **c** REC) and that were significantly associated with survival in the TNBC cases of TCGA and/or METABRIC. * indicates patients with LN- and ** with LN+

The miRNAs that were shown to present a high discriminatory power (AUC ≥0.8) in each clinical group analyzed were queried concerning survival in TNBC samples of the TCGA and METABRIC cohorts, using the KM Plot database. In the tumor size group, only one (miR-2117) out of four miRNAs, was observed in association with survival (Fig. 4a). Lower expression of this miRNA, as we observed in the group of patients with larger tumor sizes, was significantly associated with shorter survival. In the LN group, eight (miR-1225b, miR-301a, miR-378f, miR-518a, miR-520d, miR-527, miR-580; miR-595) out of 12 miRNAs were associated with survival, some of which specifically associated with LN- (miR-1225b, miR-301a, miR-520d, and miR-580) or LN+ (miR-378f, miR-527) patients (Fig. 4b). From these miRNAs, all, except miR-580a and miR-595, presented in the KM Plot, the same direction of the expression levels as we observed in our cases in the LN+ group. Finally, in the group of patients based on REC status, four out of 11 miRNAs, presented the association with survival in the TNBC samples of the TCGA and METABRIC cohorts. In this group, only miR-4536, which we observed with lower expression in the REC+ group of patients, was associated with shorter survival (Fig. 4c).

### Functional enrichment pathways

The potentially affected KEGG pathways by the differentially expressed miRNAs were determined for each of the clinical groups evaluated. The top 10 KEGG pathways ranked by *p*-value for each analysis are shown in Table 2.

**Table 2:** Top 10 KEGG pathways potentially affected by the differentially expressed miRNAs in each clinical group evaluated: tumor size, LN, and REC status (ranked by p-value)

#	KEGG pathways	***p***-value	# genes	# miRNAs
	**Tumor size (**≥**5 cm, < 5 cm)**			
1	Ubiquitin mediated proteolysis	1.46E-05	40	7
2	Circadian rhythm	0.002970639	14	7
3	Proteoglycans in cancer	0.002970639	42	7
4	Renal cell carcinoma	0.004487894	18	7
5	Signaling pathways regulating pluripotency of stem cells	0.004646529	33	6
6	Endocytosis	0.004646529	44	8
7	Glioma	0.007370446	17	7
8	Oocyte meiosis	0.015388458	25	7
9	RNA degradation	0.022814164	21	6
10	Prolactin signaling pathway	0.022814164	17	6
	**LN status**			
1	Mucin type O-Glycan biosynthesis	3.42E-10	14	8
2	Proteoglycans in cancer	6.78E-10	32	9
3	Signaling pathways regulating pluripotency of stem cells	3.68E-07	55	9
4	TGF-beta signaling pathway	3.17E-06	68	8
5	Glycosaminoglycan biosynthesis – heparan sulfate/heparin	5.72E-06	38	7
6	Hippo signaling pathway	6.54E-06	2	2
7	FoxO signaling pathway	6.94E-06	49	8
8	Circadian rhythm	2.90E-05	52	9
9	ErB signaling pathway	3.54E-05	44	9
10	Phosphatidylinositol signaling system	6.34E-05	25	7
	**REC status**			
1	Prion diseases	1.07E-05	10	8
2	Proteoglycans in cancer	1.07E-05	91	20
3	Mucin type O-Glycan biosynthesis	1.34E-05	15	12
4	Cell adhesion molecules (CAMs)	1.84E-05	65	18
5	TGF-beta signaling pathway	3.45E-05	40	16
6	Axon guidance	3.45E-05	65	16
7	Oxytocin signaling pathway	0.001164	76	18
8	N-Glycan biosynthesis	0.003947	21	15
9	Thyroid hormone signaling pathway	0.003947	53	19
10	Signaling pathways regulating pluripotency of stem cells	0.004389	65	20

The analysis of differentially expressed miRNAs, in the tumor size group, resulted in 21 pathways, including ubiquitin-mediated proteolysis (hsa04120), proteoglycans in cancer (hsa05205), and signaling pathways regulating pluripotency of stem cells (hsa04550). In the LN group, 51 KEGG pathways potentially affected by these miRNAs were identified, including mucin type-O-glycan biosynthesis (hsa00512), TGF-beta signaling pathway (hsa04350), glycosaminoglycan biosynthesis – heparan sulfate/heparin (hsa00534), hippo signaling pathway (hsa04390), ErbB signaling pathway (hsa04012). Finally, considering the REC status of the patients, 41 KEGG pathways were identified potentially affected by these miRNAs, including proteoglycans in cancer (hsa05205), cell adhesion molecules (CAMs) (hsa04514), N-Glycan biosynthesis (hsa00510), and thyroid hormone signaling pathway (hsa04919). The thyroid hormone signaling pathway was predicted to be affected by eight miRNAs (miR-1200, miR-1271-3p, miR-449b-5p, miR-4536-3p, miR-542-3p, miR-548n, miR-593-3p and miR-595) indicating that this signaling pathway may be important in the development of breast cancer recurrence.

Considering all the miRNAs selected from the three comparisons among the three clinical groups, 15 miRNAs were found to be involved in the Integrated Breast Cancer Pathway, potentially targeting 133 genes, some of them targeted by more than one miRNA (Table S2).

### In silico functional analysis and miRNA/mRNA target networks

To further elucidate the potential biological impact of each differentially expressed miRNA in the clinical groups evaluated, experimentally validated interactions between miRNA and their target genes were assessed (only strong evidence of interaction was considered for this analysis). Thirty-three of the 58 selected miRNAs (2/8 for the tumor size group, 13/23 for LN status and 18/27 for REC status) presented interactions experimentally validated, regulating a total of 295 target genes (Table S3). When considering only miRNAs with high power to discriminate among the cases in each clinical group (based on ROC analysis), a total of 13 miRNAs were observed (one for the tumor size group, seven for LN status and five for REC status) (Table 3).

**Table 3:** MiRNAs and corresponding target genes identified in each clinical group evaluated (only miRNAs with high discriminatory power (AUC ≥0.8) were included)

Clinical groups	miRNAs	***Target Genes***
**Tumor size** (≥5 cm/< 5 cm)	miR-519c-3p	*HIF1A, ABCG2, ELAVL1, TIMP2, PTEN, CDKN1A*
**LN status**	let-7f-5p	*KLK10, KLK6, PRDM1, IL3, CYP19A1, COPS8, GPS1, CCND1, COPS6, MYH9, SOCS3, ELF4, CCL7, AGO1, IL6, POSTN*
	miR-200c-3p	*TUBB3, BMI1, GEMIN2, BAP1, ZEB2, ZEB1, FN1, ZFPM2, PTPN13, RNF2, RCOR3, BRD7, ACVR2B, MSN, NTRK2, ERRFI1, CCNE2, XIAP, BCL2, TIMP2, FBLN5, VEGFA, NCAM1, IKBKB, FLT1, KLF9, TBK1, PMAIP1, NTF3, LPAR1, EDNRA, RHOA, KLHL20, PTPRD, ELMO2, ERBIN, WDR37, VAC14, TCF7L1, RASSF2, HOXB5, RIN2, KLF11, SEPT7, SHC1, MYB, ETS1, DUSP1, USP25, EFNA1, RND3, DNMT3A, DNMT3B, SP1, CFL2, CDH11, SEC23A, KDR, HFE, DLC1, ATRX, ZNF217, BTC, ZFPM1, PIN1, KRAS, NOTCH1, GATA4, SUZ12, ROCK2, UBQLN1, E2F3, MALAT1, CDK2, PRKCZ, NOS3, SIRT1, FOXO1, PDCD10, ADAM12, PTEN, LEPR, CRKL, MYLK, SH3PXD2A, DNAJC3, JAZF1, RPS6KB1, SLC1A2*
	miR-301a-5p	*PTEN, BTG1, NDRG2*
	miR-520d-5p	*PPIB*
	miR-518a-5p	*MCL1, PIK3C2A, CCL2*
	miR-580-3p	*TWIST1*
	miR-595	*PARD6A*
**REC status**	miR-184	*AKT2, INPPL1, NFATC2, SOX7, AGO2, MYC, BCL2, EZR, SND1, GAS1, ZFPM2, PDGFB, PLPP3, AKT1, BIN3, PRKCB, PPP1R13L, TNFAIP2, PKM*
	miR-449b-5p	*SIRT1, CCNE2, MET, GMNN, HDAC1, CDC25A, CDK6, MYCN, NEAT1*
	miR-542-3p	*BIRC5, ILK, MTDH, PIM1, AKT1, BMP7, RPS23, ANGPT2, OTUB1, IGFBP1, CTTN, PIK3R1, FZD7*
	miR-593-3p	*CDC274, PROP1*
	miR-595	*PARD6A*

**Fig. 5:**
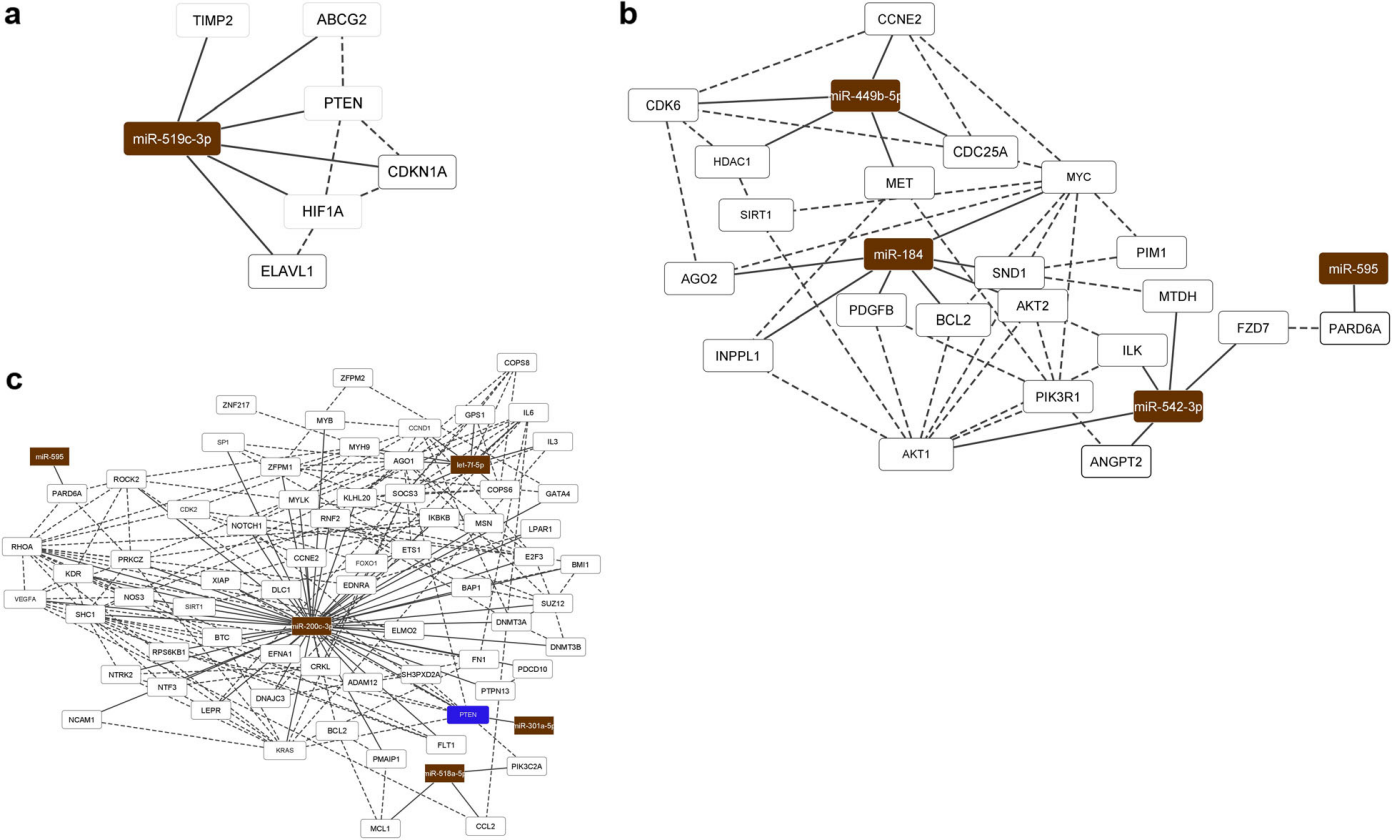
MiRNA/mRNA networks of the differentially expressed miRNAs observed in the tumor size (**a**), LN (**b**) and REC (**c**) clinical groups and their corresponding experimentally validated target genes (Cytoscape v. 3.8.0). Brown rectangle: miRNAs, white rectangles: genes targeted by one miRNA, blue rectangles: genes targeted by two miRNAs. True lines: protein-protein interaction (STRING database), doted lines: miRNA-target gene interaction

Protein-protein interactions (PPI) were evaluated considering the selected validated miRNA target genes for each clinical group and used to construct a miRNA/mRNA network (Fig. 5). For the tumor size, one network was generated based on the six target genes of miR-519c-3p (Table 3, Fig. 5a); considering their involvement in the biological process (GO), four of these targets were reported as related to negative regulation of growth (*PTEN, CDKN1A, HIF1A*), of the mitotic cell cycle (*PTEN, CDKN1A, TIMP2*), and vascular smooth muscle cell proliferation (*PTEN, CDKN1A*).

PPI analysis for the LN group was generated considering the 113 experimentally validated target genes of seven miRNAs (Table 3). Considering PPI with the highest confidence (interaction score ≥ 0.9000) and removing nodes without connections, a network was generated with 68 target genes of five miRNAs (Fig. 5b).

For the REC comparison, 41 experimentally validated target genes of five miRNAs (Table 3) were used for the PPI analysis. The miRNA/mRNA network was constructed considering the same settings as the LN network (interaction score ≥ 0.900, nodes without connection were hidden), resulting in a network with 21 genes and four miRNAs (Fig. 5c).

**Fig. 6:**
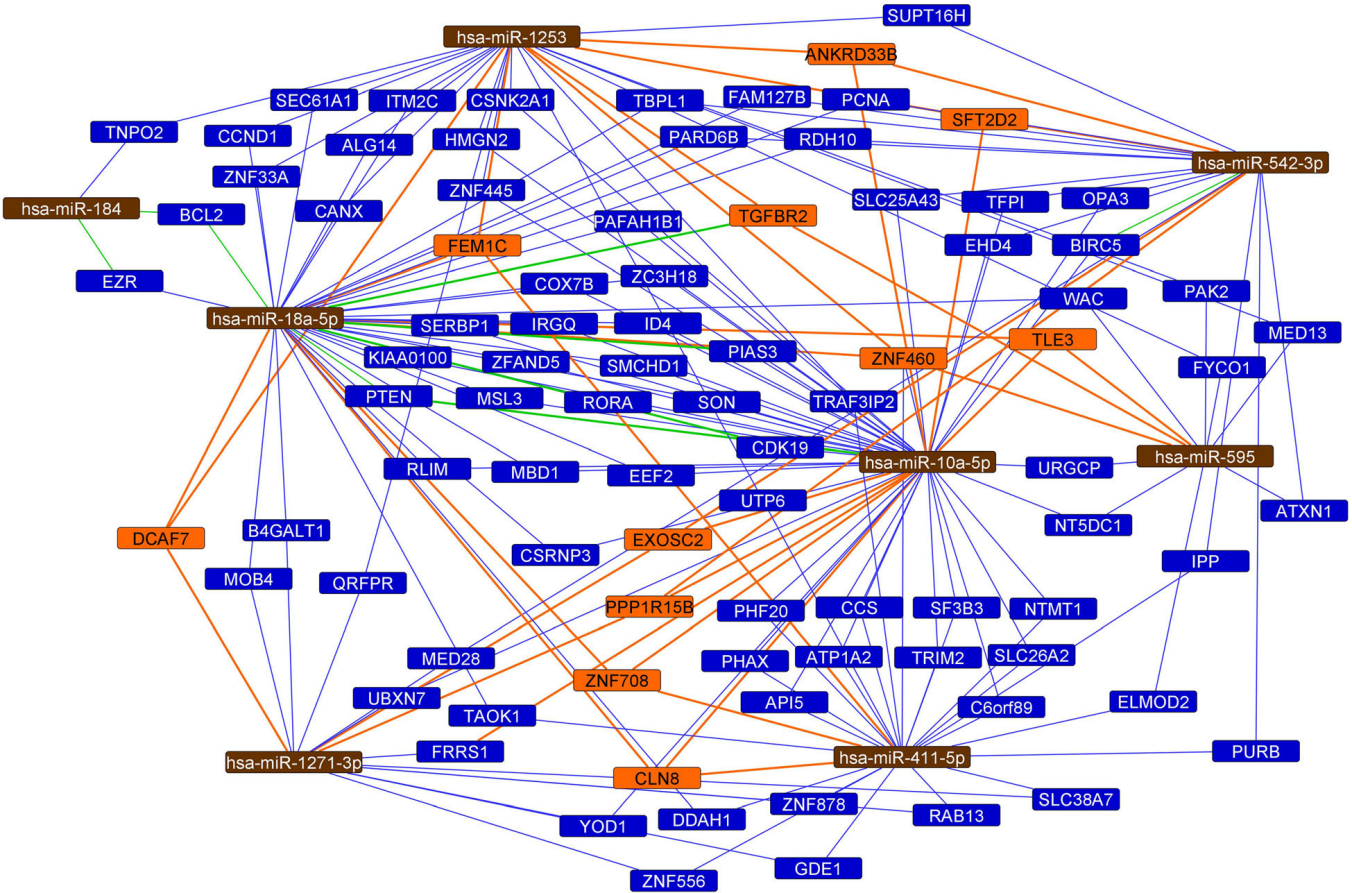
MiRNA/mRNA network of the 8 miRNAs commonly observed in the to LN/REC, LN and REC groups’ comparisons (miRTargetLink). Brown rectangle: miRNAs, blue rectangles: genes targeted by two miRNAs, orange rectangle: genes targeted by three or more miRNAs, blue and orange lines: weak evidence, green lines: strong evidence

Finally, considering the eight miRNAs common to LN/REC, LN and REC comparisons and the miRTarbase data (Fig. 6), a network was generated with 89 genes experimentally validated as a target of the 8 selected miRNAs. Most of them were validated through weak evidence of interaction, however strong evidence of interaction was also reported for miR-10a and *PTEN*, miR-184 and *BCL2* and *EZR*, miR-18a-5p and *EZR, PTEN, TGFBR2, CDK19, PIAS3,* and *BCL2,* and miR-542-3p and *BIRC5*.

To determine the interference of all differentially expressed miRNAs obtained in our analysis in the process related to breast tumorigenesis, a network was constructed considering only experimentally validated target genes presented in The Integrated Breast Cancer pathway (Table 4, Figure S2). In this network, several key oncogenes and tumor suppressor genes were found to be targeted by at least one of the differentially expressed miRNAs in our clinical group comparisons, indicating the importance of these miRNAs in breast tumor initiation and progression, affecting cell proliferation, migration and invasion capacity, and response to treatment.

**Table 4:** MiRNAs and experimentally validated target genes involved in the Integrated Breast Cancer Pathway (Wikipathways) and their involvement in the clinical groups of this study

miRNAs	Experimentally validated targets	Clinical groups
miR-1268a	*CDC25B, MAPK1, RAP1A*	LN status
miR-1268b	*CDC25B, MAPK1, RAP1A*	LN status
miR-130a-3p	*ESR1, MYC, PTEN, SMAD4, TGFBR2*	REC status
miR-184	*AKT1, BCL2, MYC*	REC status
miR-18a-5p	*ATM, BCL2, CCND1, ESR1, PTEN, RAP1A, SMAD2, SMAD4, TFGBR2, TP53*	REC status
miR-18b-5p	*ESR1, MDM2, SMAD2*	REC status
miR-200c-5p	*CDH1, MDM2, PTEN*	LN status
miR-449-5p	*CDC25A, FOSL1, HDAC1, SIRT1, SMAD4, TGFBR2*	REC status
miR-452-5p	*BMPR2, IRS1, KRAS, SMAD4*	Tumor size
miR-519a-3p	*PTEN, RB1*	REC status
miR-99b-5p	*CHEK1, MTOR, SP1*	REC status

## Discussion

In TNBC several miRNAs were observed with deregulated expression presenting with major roles in cancer progression [44, 45]. Most of the studies reporting on the miRNA expression in TNBC do not focus on minority groups or biological disparities based on the race of the patients. In fact, a search on PubMed database using the words “African American” OR “African women” OR “biological disparity” AND microRNA OR microRNAs OR miRNA OR miRNAs resulted in 83 studies related to AA women and microRNAs with only six of them with TNBC patients, which included the previous study of our group [24].

In this study, we report on the global miRNA profiling of genomically ancestral characterized AA patients with TNBC stratified in three clinical groups of patients based on tumor size, LN metastasis and breast cancer recurrence status.

Tumor size is one of the most important prognostic determinants for breast cancer [46]. Larger tumors can be a result of late diagnosis, high proliferation rate, lack of treatment, or poor response to neoadjuvant treatment. In this study, the average tumor size in the non-treated TNBC patients was 5.86 ± 5.17 cm; six of these patients were diagnosed with tumors larger than 5 cm, being categorized as at least Stage IIB (no regional LN metastasis and no clinical or radiological evidence of distant metastasis), which as expected, presents lower 5-year survival rates when compared to patients with smaller tumors [47]. Eight miRNAs were found differentially expressed between the groups of patients based on tumor size, among them, the miR-2117, miR-378c, miR519c-3p and miR-934 presented high power (AUC ≥ 0.8) to discriminate the patients in this group. Among these miRNAs, the expression of miR-2117 was observed in colorectal cancer inversely correlated with the expression levels of the target gene *TGFBR1* [48]. This gene is found overexpressed in breast cancer [49] and is involved in the MAPK-signaling pathway [48]. Downregulation of miR-378c was observed in head and neck squamous cell carcinoma (HNSCC) and appeared as one of the most important prognostic variables for HNSCC [50]. However, in our study, we observed lower levels of miR-519c-3p from patients diagnosed with larger tumors (≥5 cm). Increased levels of miR-519c-3p were found to promote tumor growth and proliferation in hepatocellular carcinoma cells, by targeting the *BTG3* gene [51]. Increased expression of miR-934 was previously reported on TNBC samples compared to ER+ tumors, however, its expression level was not associated with tumor size [52].

Lymph node (LN) status is still one of the strongest prognostic factors in breast cancer [53]. In this group, 23 miRNAs were found differentially expressed between the LN+ and LN- patients, among them, miR-1253, miR548l and mIR-873-5p, indicating that these miRNAs might be involved in conferring the invasion ability to the tumor cells. A combination of seven of these miRNAs (let-7f-5p, miR-1255b-5p, miR-1268b, miR-200c-3p and miR-520d-5p + miR-527 + miR-518a-5p), presented a more robust discriminatory power (AUC ≥ 0.9). It is important to mention, however, that miR-520d, irrespectively of the LN status, showed no differences in overall survival in the analysis of the Kmplot database. Interestingly, altered expression levels of miR-1253 and miR-548 l were reported to interfere in the migration and invasion capacity of non-small cell lung cancer (NSCLC). Altered expression of miR-548 l and miR-1253 was negatively associated with LN metastasis in NSCLC, by targeting the *AKT1* and *WNT5A* genes, respectively [54, 55]. In our study, both of these miRNAs were observed with lower expression levels in the LN+ group, indicating as in NSCLC, a similar tumor suppressor activity in breast cancer. The long non-coding RNA DCST1-AS1 was reported to act as a “sponge” by binding miR-873-5p, resulting in the upregulation of other target genes such as *IGF2BP1, MYC, LEF1,* and *CDD4* and conferring the increase of cell proliferation and metastasis capacity in TNBC cells [56]. The lower expression levels of this miRNA in the LN+ group of our study could be among other mechanisms, a consequence of high levels of this lncRNA.

In relation to recurrence (REC), 27 miRNAs were observed differentially expressed between REC+ and REC- groups, with 12 presenting an AUC value higher than 0.8; a combined analysis of three of these miRNAs (miR-1200, miR-1249-3p, and miR-1271-3p), presented a robust power (AUC ≥ 0.9) in discriminating the patients based on REC status. Deregulated expression levels of miR-1249-3p in breast cancer cells were associated with interference of lncRNA MIF-AS1 [57]. This lncRNA acts as a sponge resulting in lower expression levels of miR-1249-3p which impedes its interaction with the *HOXB8* target gene. In vitro upregulation of miR-1249-3p resulted in suppression of proliferation and migration activity and reversion of the Epithelial-Mesenchymal Transition (EMT) progress, indicating a tumor suppressor role of this miRNA in breast tumors, and corroborating with our findings that showed lower expression levels of miR-1249-3p in the REC+ group. In gliomas and osteosarcomas, it was observed that the downregulation of miR-1200, led to the up-regulation of the *HOXB2* gene, which increased proliferation and invasion capacity of the tumor cells [58, 59]. These data indicate a tumor suppressor activity of miR-1200, that as miR-1249-3p, presented lower expression levels in the REC+ group. Finally, for miR-1271, to our knowledge, there is no reported deregulation of its expression in tumor cells.

TNBC patients can show satisfactory response to chemotherapy, especially in the neoadjuvant setting [60–62]. However early recurrence is more frequent in this breast cancer subtype when compared to others, which usually occurs within the first 3 years after diagnosis [5, 63]. Chemotherapy adherence and uptake have also been shown to differ among patients’ racial/ethnic groups [64–67]. In TNBC, a previous study conducted by our group [68], showed however that although a substantial number of TNBC patients failed to receive and/or complete chemotherapy, AA patients presented a higher chemotherapy uptake than White patients. Interestingly, and aware of the limitation of the sample size of this present study, seven patients presented recurrence after treatment while fifteen patients were disease-free. Considering both LN and REC status, 22 miRNAs were found differentially expressed among the LN+/REC+, LN+/REC-, LN−/REC+, and LN−/REC- groups, eight of them also presented in the comparison of LN and REC clinical groups comparisons: miR-10a-5p, miR-1253, miR-1271-3p, miR-184, miR-18a-5p, miR-411-5p, miR-542-3p, and again miR-595. Finally, a query of the KM Plot database of the TCGA and METABRIC TNBC cases, showed that several of the miRNAs observed with deregulated expression in the patients with larger tumor size, positive LN and REC were significantly associated with lower survival rates. It is important to mention, however, that some miRNAs which expression levels were associated with REC of the patients, in the KM Plot showed higher survival rates. Considering that this database, which compiles cases from the TCGA and Metabric breast cancer patients, may not reflect the clinical and biological characteristics of our AA patients, as well as their socio-economic condition. The latter largely impacts health access and ultimately overall survival rates. In addition, the differences in specimen types, miRNA profiling, and other technical variables can largely affect miRNA expression levels.

Interestingly, the involvement of 12 of the miRNAs observed in the TNBC cases of our study and that were differentially expressed in the above clinical groups was among the ones observed with differential expression in our previous study in TNBC cases of AA and non-Hispanic White (NHW) patients [24]. Among these common miRNAs, 12 of them presented the same level of expression of this study, two of which associated with tumor size (miR-2117 and miR-617), eight with LN status (miR-1253, miR-1268a, miR-200c-3p, miR-520d-5p, miR-518a-5p, miR-528, miR-580 and miR-873-5p), and two with REC status (miR-1200 and miR-449b-5p). These associations could indicate that these 12 miRNAs are intrinsically regulated in the TNBC of AA patients, and could account for the observed more aggressive phenotype of their tumors when compared to the NHW patients. This suggestion can be supported by the analysis of the KEGG pathway of the differentially expressed miRNAs observed in each clinical group of this study, which revealed their involvement in signaling pathways often associated with tumor aggressiveness. In the LN group, for example, considering only the panel of seven miRNAs that presented the best capability to discriminate LN+ and LN- cases, among the top ten KEGG pathways observed, three were affected by six of these miRNAs: signaling pathways regulating pluripotency of stem cells, TGF-beta and Hippo signaling pathways. In the REC group, among the top ten KEGG pathways, the thyroid hormone signaling pathway was potentially affected by eight of the 12 miRNAs in the highest discriminatory panel (miR-1200, miR-1271-3p, miR-449b-5p, miR-4536-3p, miR-542-3p, miR-548n, miR-593-3p and miR-595). This signaling pathway can regulate tumor progression and apoptosis process through thyroid hormones (TR) genomic and non-genomic actions [69]. TR expression was associated with higher 5-year survival in metastasized *BRCA1* mutated breast cancer [70]. *BRCA1-*mutation carriers frequently develop TNBC [71], and a disturbance of this signaling pathway may interfere with the survival of TNBC patients. Considering these miRNAs in the above clinical groups, miRNA/mRNA pairings that were experimentally validated showed a number of targets that play relevant roles in breast cancer progression, including genes that are part of the subclassification of the TNBC into the six molecular subtypes [72] among them, genes involved in the epithelial-to-mesenchymal transition (EMT) process, which is essential to confer cell migration and invasion and stem cell tumor capabilities [73, 74], and which miRNAs are important regulators [75, 76]. Of note, one of the most prevalent TNBC subtypes in AA patients, the mesenchymal-like (ML) subtype [77–79], is enriched in genes involved in the regulation of EMT and in the biology of cancer stem cells, both markers that confer clinical aggressiveness [80, 81]. In addition, some of the differentially expressed miRNAs observed in our study are highly involved in breast cancer progression as shown by their predicted interactions with relevant genes of the Integrated Breast Cancer Pathway. Some of these interactions were already experimentally validated, including miR-184 and *AKT1K, BCL2 and MYC,* and miR-200c, miR-130a-3p, miR-18a-5p, miR-519a-3p and *PTEN*. These interactions indicate an important role for these miRNAs in the tumorigenesis of TNBC in AA patients, which should be further explored in other independent well-characterized and large AA populations.

## Conclusion

Altogether, our data indicate that alterations in miRNA expression are relevant biological factors that can be associated with the poor prognosis in TNBC of AA patients, by conferring to their TNBC cells, increase cell proliferation, elevate expression of angiogenesis markers and/or high migration and invasive cellular capabilities that are reflected in the clinical characteristics evaluated in this study. Therefore, apart from the socioeconomic and cultural factors that play a significant role in the observed disparities in incidence and mortality rates in TNBC of AA women, miRNAs deregulation can also be included as drivers of these disparities.

## Supplementary Information

**Additional file 1: Table S1.** Summary of the clinical and histopathological characteristics of the TNBC-AA patients analyzed in this study.

**Additional file 2: Figure S1.** Principal Component Analysis (PCA) showing the clustering of the AA patients (black dots) with the Africans, African Americans and African Caribbean groups based on genotype analysis. The horizontal axis represents Principal Component 1 (PC1) and vertical axis Principal Component 2 (PC2).

**Additional file 3: Table S2.** MiRNAs that are significantly enriched and targets in Integrated Breast Cancer Pathway in each clinical group evaluated. (*P* < 0.05).

**Additional file 4: Table S3.** Experimentally validated target genes of 33 selected miRNAs differentially expressed in the three group comparisons. The Integrated Breast Cancer Pathway with miRNAs and experimentally validated target genes. Tumor size related miRs and target genes (pink), LN related miRs and target genes (orange), REC related miRs and target genes (blue), target genes associated with more than one comparison (green). Red lines represent inhibitory interaction, green lines represent stimulatory interaction, and black lines represent miRNA interaction with target.

**Additional file 5: Figure S2.** The Integrated Breast Cancer Pathway with miRNAs and experimentally validated target genes. Tumor size related miRs and target genes (pink), LN related miRs and target genes (orange), REC related miRs and target genes (blue), target genes associated with more than one comparison (green). Red lines represent inhibitory interaction, green lines represent stimulatory interaction, and black lines represent miRNA interaction with target.

## Data Availability

The datasets generated and analyzed during the current study are available in the “Data repository for Manuscript: A Panel of miRNAs as Prognostic Markers for African-American Patients with Triple Negative Breast Cancer” (https://app.box.com/s/9h5wfr95ixqjrcjvjm26ul5lg73w8kbv).

## Abbreviations

AA: African-American
TNBC: Triple negative breast cancer
LN: Lymph node
REC: Recurrence
ROC: Receiver Operating Characteristic
miRNAs: microRNAs
ER: Estrogen receptor
PR: Progesterone receptor
HER2: Human epidermal growth factor receptor-2
TCGA: The Cancer Genome Atlas
METABRIC: Molecular Taxonomy of Breast Cancer International Consortium
PPI: Protein-protein interactions
GO: Biological process
HNSCC: Head and neck squamous cell carcinoma
NSCLC: Non-small cell lung cancer
EMT: Epithelial-Mesenchymal Transition
NHW: Non-Hispanic White
ML: Mesenchymal-like
