# Supplementary material for: A panel of miRNAs as prognostic markers for African-American patients with triple negative breast cancer
Source: BMC Cancer. 2021 Jul 27;21:861. doi: 10.1186/s12885-021-08573-2 (PMC8317413; doi:10.1186/s12885-021-08573-2)
Supplement: Supplementary file 1 — Additional file 1: Table S1. Summary of the clinical and histopathological characteristics of the TNBC-AA patients analyzed in this study. [file 12885_2021_8573_MOESM1_ESM.doc]

**Table S1.** Summary of the clinical and histopathological characteristics of the TNBC-AA patients analyzed in this study.

| **Characteristics** | **n(%)/range** |
| --- | --- |
| **Mean age** | 55.27±9.96 / 32-78 years |
| **Histological type** |  |
| Invasive Ductal Carcinoma NOS | 18 (81.8%) |
| Comedocarcinoma | 1 (4.55%) |
| Cribiform carcinoma | 1 (4.55%) |
| Mucin-producing adenocarcimoma | 1 (4.55%) |
| Micropapillary ductal carcinoma | 1 (4.55%) |
| N/A | 3 |
| **Mean tumor size** | 5.86±5.17cm / 1.1-22cm |
| < 5 cm | 10 (62.5%) / 1.1-3.8 cm |
| ≥ 5 cm | 6 (37.5%)/ 5.0-22cm |
| N/A | 9 |
| **Lymph node status** |  |
| POS | 10 (47.6%) |
| NEG | 11 (53.4%) |
| N/A | 4 |
| **Recurrence status** |  |
| POS | 7 (32%) |
| NEG | 15 (68%) |
| N/A | 3 |
| **Histological grade** |  |
| Grade 2 | 1 (4%) |
| Grade 3 | 24 (96%) |

NOS: no otherwise specified, N/A: not available
