# Supplementary figures and images for: A panel of miRNAs as prognostic markers for African-American patients with triple negative breast cancer
Source: BMC Cancer. 2021 Jul 27;21:861. doi: 10.1186/s12885-021-08573-2 (PMC8317413; doi:10.1186/s12885-021-08573-2)

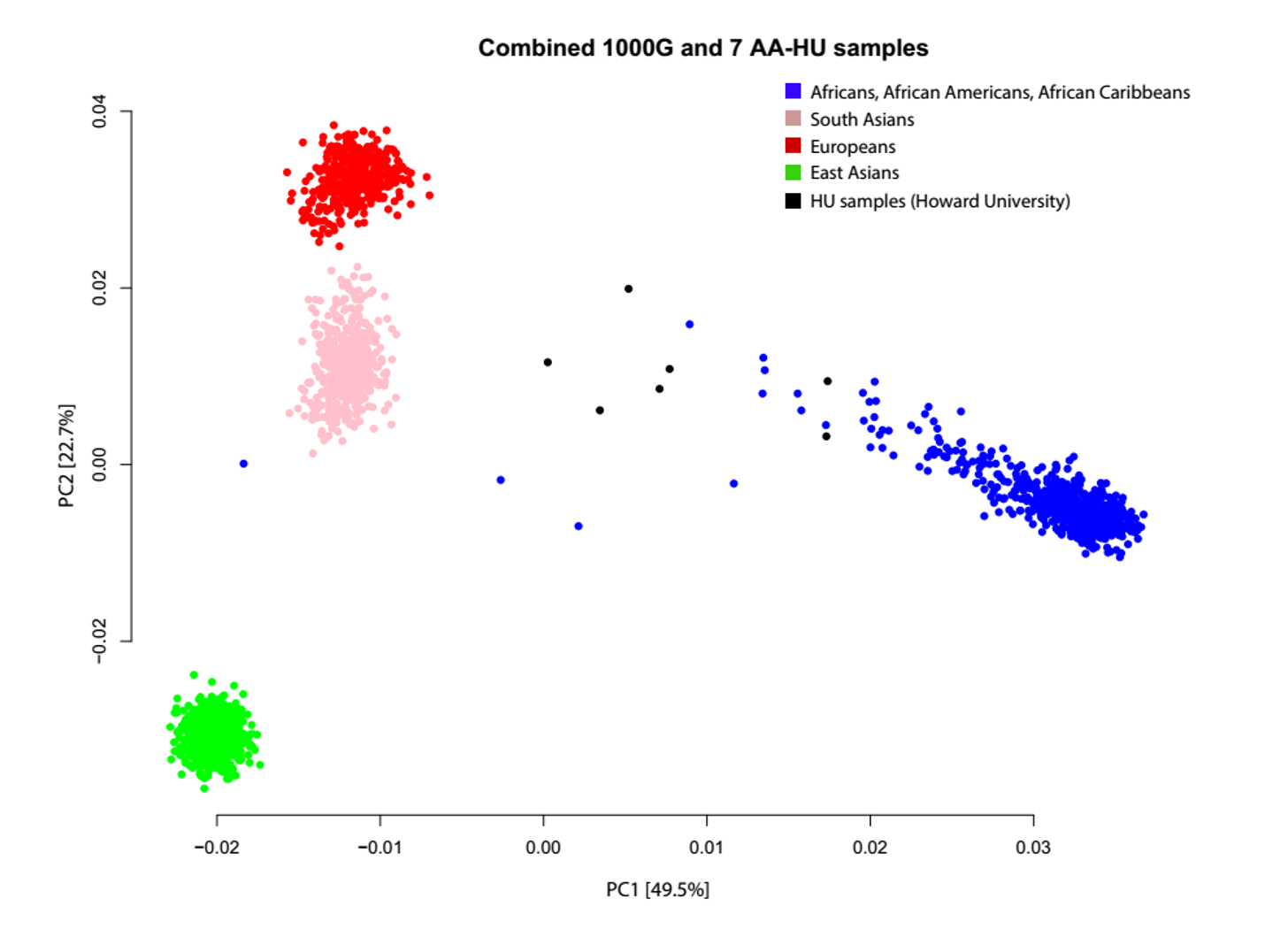

Supplement: Supplementary file 2 — Additional file 2: Figure S1. Principal Component Analysis (PCA) showing the clustering of the AA patients (black dots) with the Africans, African Americans and African Caribbean groups based on genotype analysis. The horizontal axis represents Principal Component 1 (PC1) and vertical axis Principal Component 2 (PC2). [file 12885_2021_8573_MOESM2_ESM.tif]

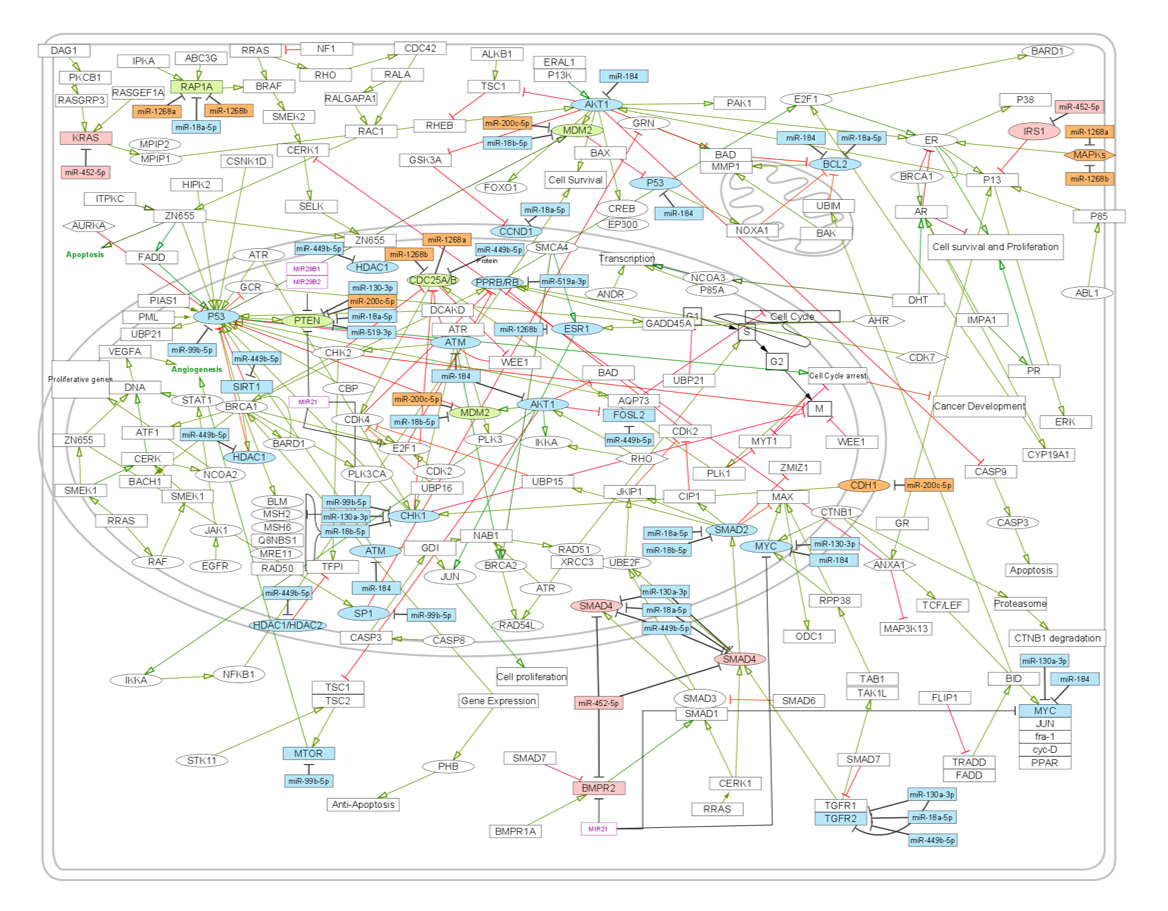

Supplement: Supplementary file 5 — Additional file 5: Figure S2. The Integrated Breast Cancer Pathway with miRNAs and experimentally validated target genes. Tumor size related miRs and target genes (pink), LN related miRs and target genes (orange), REC related miRs and target genes (blue), target genes associated with more than one comparison (green). Red lines represent inhibitory interaction, green lines represent stimulatory interaction, and black lines represent miRNA interaction with target. [file 12885_2021_8573_MOESM5_ESM.tif]
