# Supplementary material for: A panel of miRNAs as prognostic markers for African-American patients with triple negative breast cancer
Source: BMC Cancer. 2021 Jul 27;21:861. doi: 10.1186/s12885-021-08573-2 (PMC8317413; doi:10.1186/s12885-021-08573-2)
Supplement: Supplementary file 3 — Additional file 3: Table S2. MiRNAs that are significantly enriched and targets in Integrated Breast Cancer Pathway in each clinical group evaluated. (P < 0.05). [file 12885_2021_8573_MOESM3_ESM.doc]

**Table S2.** **MiRNAs that are significantly enriched and targets in Integrated Breast Cancer Pathway in each clinical group evaluated. (P < 0.05)**

| **Clinical groups** | **miRNAs** | ***Target genes*** |
| --- | --- | --- |
| **Tumor size**  (≥5 cm/<5 cm) | miR-452-5p | *BMPR2, IRS1, KRAS, SMAD4* |
|  | miR-5196-5p | *ABL1,AR,ARAF,ATM,BACH1,BAD,BAK1,BCL2,BID,BMPR1A,BMPR2,BRCA1,CASP9,CCND1,CDC25A,CDC25B,CDC42,CDH1,CDK2,CDK4,CERK,CHEK1,CHUK,CREB1,CSNK1D,CYP19A1,DAG1,DCAKD,E2F1,EGFR,EP300,ESR1,FER,FILIP1,FOSL1,FOXO1,GDI1,GRN,GSK3A,HDAC1,HIPK2,HMGCR,IMPA1,IRS1,KRAS,MAP3K13,MAPK1,MAX,MRE11,MSH2,MYT1,NAB1,NF1,PAK1,PHB,PIAS1,PIGR,PIK3R2,PKIA,PLK1,PLK3,PML,PTEN,RAD50,RALGAPA1,RAP1A,RASGEF1A,RASGRP3,RHO,SMAD2,SMAD6,SMAD7,SP1,STAT1,STK11,TAB1,TFPI,TGFBR1,TGFBR2,TP53,TRADD,TSC1,UBE2F,USP15,USP38,VEGFA,WEE1,XRCC3,ZMIZ1,ZMYND8,ZNF655* |
| **LN status** | miR-1253 | *ABL1,AHR,ALKBH1,ANXA1,AR,ATM,BACH1,BAK1,BARD1,BCL2,BID,BLM,BMPR1A,BMPR2,BRCA1,CASP9,CCND1,CDH1,CDK2,CDK4,CERK,CHEK1,CHEK2,CHUK,CREB1,CSNK1D,CYP19A1,DAG1,DCAKD,DHTKD1,E2F1,EGFR,ESR1,FER,FILIP1,FOSL1,FOXO1,HDAC1,HIPK2,HMGCR,IMPA1,IRS1,ITPKC,JAK1,JUN,MAP3K13,MAP3K7CL,MAPK1,MAX,MRE11,MSH2,MSH6,NAB1,NF1,PAK1,PHB,PIGR,PIK3R2,PKIA,PML,PPP4R3A,PTEN,RAD50,RAD51,RALA,RAP1A,RASGRP3,RB1,RHO,SMAD1,SMAD2,SMAD7,SP1,STAT1,TAB1,TFPI,TGFBR2,TP53,TPR,TRADD,TSC1,USP15,USP38,WEE1,XRCC3,ZMIZ1,ZNF655* |
|  | miR-1268a | *CDC25B,MAPK1,RAP1A* |
|  | miR-1268b | *CDC25B,MAPK1,RAP1A* |
|  | miR-200c-3p | *BCL2,CDK2,EP300,FOXO1,IRS1,JUN,KRAS,PTEN,SIRT1,SP1,VEGFA* |
|  | miR-513b-5p | *ABL1,AHR,ALKBH1,AR,ATM,BACH1,BAK1,BARD1,BCL2,BID,BMPR1A,BMPR2,CASP3,CASP9,CCND1,CDC42,CHEK1,CHUK,CREB1,CSNK1D,CTNNB1,DAG1,DHTKD1,EGFR,ESR1,FADD,FER,FILIP1,FOSL1,FOXO1,HDAC1,HIPK2,HMGCR,IMPA1,IRS1,JAK1,JUN,KRAS,MAP3K13,MAPK1,MAX,MRE11,MSH2,MSH6,MYCBP2,MYT1,NAB1,NF1,NUP85,ODC1,PAK1,PIK3R2,PKIA,PPP4R3A,PTEN,RAC1,RAD51,RALA,RASGRP3,RHO,RPP38,SIRT1,SMAD2,SMAD6,SP1,TAB1,TFPI,TGFBR1,TGFBR2,TP53,TPR,TSC1,UBE2F,USP15,USP21,USP38,WEE1,ZMIZ1,ZMYND8,ZNF655* |
|  | miR-548l | *ABL1,AHR,ALKBH1,APOBEC3G,AR,ARAF,ATF1,ATM,BACH1,BAK1,BARD1,BAX,BCL2,BID,BMPR1A,BMPR2,CASP3,CCND1,CDK4,CERK,CHEK1,CHUK,CREB1,CSNK1D,CTNNB1,CYP19A1,DAG1,DCAKD,DHTKD1,EGFR,EP300,ESR1,FER,FILIP1,FOSL1,FOXO1,HDAC1,HIPK2,HMGCR,IMPA1,IRS1,JAK1,KRAS,MAP3K13,MAPK1,MAX,MRE11,MSH2,MSH6,MYCBP2,NAB1,NF1,NFKB1,PAK1,PHB,PIAS1,PKIA,PPP4R3A,PTEN,RAC1,RAD50,RAD51,RALA,RAP1A,RASGEF1A,RASGRP3,RB1,SIRT1,SMAD1,SMAD2,SMAD6,SMAD7,SP1,STAT1,TAB1,TFPI,TGFBR1,TGFBR2,TPR,TRADD,TSC1,UBE2F,USP15,USP16,USP38,WEE1,XRCC3,ZMIZ1,ZMYND8,ZNF655* |
| **REC status** | miR-130a-3p | *ESR1,MYC,PTEN,SMAD4,TGFBR2* |
|  | miR-184 | *AKT1,BCL2,MYC* |
|  | miR-18a-5p | *ATM,BCL2,ESR1,PTEN,SMAD2,SMAD4,TGFBR2* |
|  | miR-411-5p | *ABL1,AHR,ALKBH1,AR,ATF1,ATM,BAK1,BARD1,BCL2,BID,BMPR1A,BMPR2,BRCA1,CASP3,CASP9,CDH1,CDK4,CERK,CHEK1,CHUK,CREB1,CSNK1D,CYP19A1,DHTKD1,EGFR,ESR1,FER,FILIP1,FOXO1,GADD45A,GSK3A,HIPK2,IMPA1,IRS1,ITPKC,MAP3K13,MAPK1,MAX,MMP1,MRE11,MSH2,MSH6,NAB1,NF1,PAK1,PHB,PIAS1,PIK3R2,PKIA,PPP4R3A,PTEN,RAD51,RALA,RAP1A,RASGEF1A,RASGRP3,SIRT1,SMAD1,SMAD2,SMAD6,SP1,TAB1,TFPI,TGFBR2,TPR,TSC1,UBE2F,USP15,WEE1,ZMIZ1,ZMYND8,ZNF655* |
|  | miR-449b-5p | *CDC25A,HDAC1,SIRT1* |
|  | miR-548n | *ABL1,AHR,ALKBH1,AR,ARAF,ATF1,ATM,BACH1,BARD1,BAX,BCL2,BID,BLM,BMPR1A,BMPR2,CASP3,CASP9,CCND1,CDH1,CDK2,CDK4,CDK7,CERK,CHEK1,CHEK2,CHUK,CREB1,CTNNB1,CYP19A1,DAG1,DCAKD,DHTKD1,EGFR,EP300,ESR1,FER,FILIP1,FOSL1,FOXO1,HDAC1,HIPK2,HMGCR,IMPA1,IRS1,JAK1,JUN,KRAS,MAP3K13,MAP3K7CL,MAPK1,MAX,MRE11,MSH2,MSH6,MYCBP2,MYT1,NAB1,NF1,NFKB1,NOXA1,PAK1,PHB,PIAS1,PIGR,PKIA,PLK3,PML,PPP4R3A,PTEN,RAC1,RAD50,RAD54L,RALA,RALGAPA1,RAP1A,RASGEF1A,RASGRP3,RB1,SIRT1,SMAD1,SMAD2,SMAD6,SP1,STAT1,TAB1,TFPI,TGFBR1,TGFBR2,TP53,TPR,TSC1,UBE2F,USP15,USP16,USP21,USP38,VEGFA,WEE1,XRCC3,ZMIZ1,ZMYND8,ZNF655* |
|  | miR-99b-5p | *CHEK1,MTOR,SP1* |
