# Supplementary material for: A panel of miRNAs as prognostic markers for African-American patients with triple negative breast cancer
Source: BMC Cancer. 2021 Jul 27;21:861. doi: 10.1186/s12885-021-08573-2 (PMC8317413; doi:10.1186/s12885-021-08573-2)
Supplement: Supplementary file 4 — Additional file 4: Table S3. Experimentally validated target genes of 33 selected miRNAs differentially expressed in the three group comparisons. The Integrated Breast Cancer Pathway with miRNAs and experimentally validated target genes. Tumor size related miRs and target genes (pink), LN related miRs and target genes (orange), REC related miRs and target genes (blue), target genes associated with more than one comparison (green). Red lines represent inhibitory interaction, green lines represent stimulatory interaction, and black lines represent miRNA interaction with target. [file 12885_2021_8573_MOESM4_ESM.doc]

**Table S3.**  Experimentally validated targets genes of 33 selected miRNAs differentially expressed in the three group comparisons.

| **Clinical groups** | **miRNAs** | ***Target genes*** |
| --- | --- | --- |
| **Tumor size**  (≥5 cm/<5 cm | hsa-miR-452-5p | *DPYSL2, KRAS, THRB, BMI1, LEF1, TCF4, CDKN1B* |
|  | hsa-miR-519c-3p | *HIF1A, ABCG2, ELAVL1, TIMP2, PTEN, CDKN1A* |
| **LN Status** | hsa-let-7f-5p | *KLK10, KLK6, PRDM1, IL13, CYP19A1, COPS8, GPS1, CCND1, COPS6, MYH9, SOCS3, ELF4, DYRK2, CCL7, AGO1, IL6, POSTN* |
|  | hsa-miR-1253 | *BTK* |
|  | hsa-miR128-1-5p | *SNAI1, WNT3A, BMI1, E2F3* |
|  | hsa-miR-133a-5p | *FSCN1, EGFR, GDNF, RHOA, MMP9* |
|  | hsa-miR-200c-3p | *TUBB3, BMI1, GEMIN2, BAP1, ZEB2, ZEB1, FN1, ZFPM2, PTPN13, RNF2, RCOR3, BRD7, ACVR2B, MSN, NTRK2, ERRFI1, CCNE2, XIAP, BCL2, TIMP2, FBLN5, VEGFA, NCAM1, IKBKB, FLT1, KLF9, TBK1, PMAIP1, NTF3, LPAR1, EDNRA, RHOA, KLHL20, PTPRD, ELMO2, ERBIN, WDR37, VAC14, TCF7L1, RASSF2, HOXB5, RIN2, KLF11, SEPT7, SHC1, MYB, ETS1, DUSP1, USP25, EFNA1, RND3, DNMT3A, DNMT3B, SP1, CFL2, CDH11, SEC23A, KDR, HFE, DLC1, ATRX, ZNF217, BTC, ZFPM1, PIN1, KRAS, NOTCH1, GATA4, SUZ12, ROCK2, UBQLN1, E2F3, MALAT1, CDK2, PRKCZ, NOS3, SIRT1, FOXO1, PDCD10, ADAM12, PTEN, LEPR, CRKL, MYLK, SH3PXD2A, DNAJC3, JAZF1, RPS6KB1, SLC1A2* |
|  | hsa-miR-301a-5p | *PTEN, BTG1, NDRG2* |
|  | hsa-miR-367-3p | *MDM2, KLF4, RAB23, FBXW7* |
|  | hsa-miR-513b-5p | *HMGB3, GNG13, DR1, BTG3* |
|  | hsa-miR-520d-5p | *PPIB* |
|  | hsa-miR-518a-5p | *MCL1, PIK3C2A, CCL2* |
|  | hsa-miR-580-3p | *TWIST1* |
|  | hsa-miR-595 | *PARD6A* |
|  | hsa-miR-873-5p | *SRCIN1, CDK3, ABCB1* |
| **REC status** | hsa-miR-10a-5p | *HOXA1, USF2, NOCOR2, MAP3K7, BTRC, SRSF1, TRA2B, EPHA4, CHL1, ACTG1, BCL2L11, PTEN, PIK3CG, SERPINE1, GP1BA, NOD2, MMP14, MAPK8IP1, BDNF, BCL6, HOXA5, MAFB, TAC1, ATXN1, MEOX2, CSF1, HOXA10, KLF4, PPARG, DICER1, ATG2B, ESR1, IFITM1, RUNX3, RAB5A, SMAD4, GJA1, PDGFRA, TNF, SLAIN1, TGFBR2, XIAP, Acvr1, IL18, TGFB1, PPARA, MYC, PTEN, DLL4, PPARGC1A, MAP3K12, MECP2* |
|  | hsa-miR-184 | *AKT2, INPPL1, NFATC2, SOX7, AGO2, MYC, BCL2, EZR, SND1, GAS1, ZFPM2, PDGFB, PLPP3, AKT1, BIN3, PRKCB, PPP1R13L, TNFAIP2, PKM* |
|  | hsa-miR-18a-5p | *ESR1, PTEN, CTGF, TNFSF11, NR3C1, HIF1A, TGFBR2, SMAD4, HSF2, ATM, NEDD9, CDK19, DICER1, SMAD3, PHLPP1, PIAS3, BCL2, TBPL1, SMAD2, SDC4, STK4, BCL2L10, FCGR2B, NEO1, DNMT1, IRF2, RUNX1, MEF2D, TNFAIP3, NR1I2* |
|  | hsa-miR-376a-5p | *SLC16A1, TTK, SRSF11, RAP2A, AMFR* |
|  | hsa-miR-411-5p | *GRB2, SPRY4* |
|  | hsa-miR-449b-5p | *SIRT1, CCNE2, MET, GMNN, HDAC1, CDC25A, CDK6, MYCN, NEAT1* |
|  | hsa-miR-491-5p | *BCL2L1, MMP9, MMP2, TP53, GIT1, SMAD3, NOTCH3, IGF2BP1, EGFR, CDK6, CAPNS1, KDM4B, WNT3A, SLC6A3* |
|  | hsa-miR-517c-3p | *PTK2B* |
|  | hsa-miR-519a-3p | *ELAVL1, YES1, DICER1, PTEN, CDKN1A, TIMP1, RB1, FOXF2, STAT3* |
|  | hsa-miR-542-3p | *BIRC5, ILK, MTDH, PIM1, AKT1, BMP7, RPS23, ANGPT2, OTUB1, IGFBP1, CTTN, PIK3R1, FZD7* |
|  | hsa-miR-587 | *PPP2R1B* |
|  | hsa-miR-593-3p | *CDC274, PROP1* |
|  | hsa-miR-595 | *PARD6A* |
|  | hsa-miR-891a-5p | *NFKBIA* |
|  | hsa-miR-99b-5p | *RAVER2, MTOR, IGF1R, ARID3A* |
|  | hsa-miR-197-3p | *FOXO3, TUSC2, NSUN5, CD82, BMF, PMAIP1, MTHFD1, FOXJ2, MAPK1, RAN* |
|  | hsa-miR-208a-3p | *CDKN1A, MED13, ETS1, SOX6, CACNA1C, CACNB2, PDCD4, Qk, QKI* |
|  | hsa-miR-362-5p | *CYLD, PIK3C2B* |
